# The silent protectors: how neutrophils influence female fertility during cancer treatment – a narrative review

**DOI:** 10.1097/MS9.0000000000003526

**Published:** 2025-07-16

**Authors:** Emmanuel Ifeanyi Obeagu

**Affiliations:** Department of Biomedical and Laboratory Science, Africa University, Zimbabwe

**Keywords:** cancer treatment, female fertility, immune response, neutrophils, oncofertility

## Abstract

Neutrophils, the most abundant white blood cells in the body, are vital in defending against infections, but they also play significant roles in various physiological and pathological conditions, including female fertility. This review examines the dual role of neutrophils in female fertility during cancer treatment, particularly focusing on how they influence ovarian function and oncofertility. Chemotherapy and radiation, commonly used in cancer treatment, induce inflammatory responses that recruit neutrophils to ovarian tissue, where they release cytokines and enzymes that may contribute to ovarian damage, leading to reduced fertility. While neutrophils are often seen as contributors to tissue damage through the release of reactive oxygen species and inflammatory cytokines, they also have protective roles. Recent studies suggest that neutrophils can promote tissue repair and regeneration by releasing growth factors that aid in the recovery of damaged ovarian tissue. These findings indicate a complex interaction between neutrophils and the ovarian microenvironment, with the potential to either enhance or impair fertility depending on the context of their activation.

**HIGHLIGHTS**

- Immunological balance: Neutrophils regulate immune tolerance, preventing excessive inflammation that could impair fertility during cancer therapy.
- Ovarian protection: They contribute to ovarian tissue repair, mitigating chemotherapy-induced damage.
- Endometrial support: Neutrophils aid in endometrial remodeling, essential for embryo implantation.
- Hormonal interplay: They interact with reproductive hormones, influencing ovarian function.
- Fertility challenges: Dysregulated neutrophil activity may exacerbate infertility risks in female cancer patients.

## Introduction

Cancer treatments such as chemotherapy, radiation, and surgery have revolutionized the survival rates of women diagnosed with cancer^[1,2]^. However, these treatments often lead to significant side effects, one of the most critical being the impact on reproductive health and fertility. Female fertility can be compromised by chemotherapy and radiotherapy, which damage ovarian follicles, disrupt hormonal signaling, and lead to premature ovarian failure^[3,4]^. In response to this, oncofertility has emerged as a specialized field that focuses on preserving fertility in women undergoing cancer treatment. While fertility preservation techniques such as oocyte cryopreservation and ovarian tissue freezing have provided viable solutions, there remains a need to understand the underlying biological mechanisms that contribute to fertility loss and how they can be mitigated^[5-7]^. The immune system, particularly the role of neutrophils, plays a vital yet often overlooked part in the ovarian function during cancer treatment. Neutrophils are the first responders to infections and are part of the innate immune system. Their primary function is to combat pathogens and initiate inflammation, but their involvement in tissue damage and repair extends beyond infection control. Recent research has suggested that neutrophils can influence ovarian health, especially in the context of cancer treatment, by releasing cytokines, reactive oxygen species (ROS), and proteases, which may contribute to ovarian damage or tissue regeneration^[8,9]^. The ovarian microenvironment is delicate and highly responsive to both external insults, such as chemotherapy, and internal immune responses. The infiltration of neutrophils into the ovaries, triggered by chemotherapy and radiation-induced inflammation, can exacerbate ovarian damage, disrupt follicular development, and cause hormonal imbalances^[10]^. These effects can result in infertility or a reduced ovarian reserve, significantly compromising fertility. On the other hand, neutrophils may also promote tissue repair and regeneration through the release of growth factors and cytokines, which suggests a more complex role than merely causing damage^[11]^.

Ovarian follicles, the basic functional units in the ovaries, are composed of oocytes surrounded by granulosa and theca cells. During cancer treatment, the ovarian follicles are highly susceptible to damage due to their rapid division and sensitivity to chemotherapy and radiation^[3]^. Neutrophils, as part of the inflammatory response, infiltrate ovarian tissue and can influence follicular function either by exacerbating tissue damage or by promoting repair^[12]^. The extent to which neutrophils contribute to ovarian dysfunction versus recovery remains an area of active investigation, as their activity may vary depending on the timing and type of cancer treatment, as well as the level of ovarian damage already present. Inflammation caused by neutrophils during cancer treatment may have lasting effects on the ovarian reserve and fertility outcomes^[13]^. The presence of pro-inflammatory cytokines such as tumor necrosis factor-alpha (TNF-α) and interleukins (e.g., IL-1β, IL-6) can accelerate follicular atresia, the process through which non-dominant follicles undergo degeneration. Moreover, neutrophils release proteases, such as matrix metalloproteinases (MMPs), which break down extracellular matrix components and can disrupt the structure of the ovarian microenvironment. This tissue remodeling, while necessary for immune responses, can interfere with normal ovarian function, further complicating fertility preservation in women undergoing chemotherapy or radiotherapy^[14]^. Despite their potential to cause harm, neutrophils also have a reparative function. After tissue damage, neutrophils can release growth factors like vascular endothelial growth factor (VEGF) and fibroblast growth factor (FGF), which are important for tissue healing and angiogenesis^[15]^. In the ovaries, neutrophils may play a role in repairing damaged ovarian tissue and promoting the regeneration of the ovarian stroma, which could mitigate the effects of chemotherapy and radiation. This reparative function may help to preserve fertility in women who experience sub-lethal ovarian damage, allowing for the possibility of fertility restoration over time^[16,17]^.

## Aim

The aim of this review is to explore the dual role of neutrophils in oncofertility, particularly in the context of cancer treatments, and to identify potential strategies for modulating neutrophil activity to protect ovarian function and preserve fertility in women undergoing cancer therapy.

## Review methods

This narrative review aimed to synthesize existing literature on the role of neutrophils in oncofertility, focusing on their dual influence on ovarian function during cancer treatments and the potential strategies for modulating their activity to preserve fertility. The review followed a systematic process of gathering, analyzing, and synthesizing current research from various sources, including peer-reviewed journal articles, clinical studies, and preclinical models, to provide an overview of the subject. The methods used to conduct this review are described below.

## Literature search and selection criteria

A comprehensive literature search was conducted using several academic databases, including PubMed, Google Scholar, and Scopus, with the aim of identifying relevant articles published in the last two decades. Keywords such as “neutrophils,” “oncofertility,” “ovarian function,” “chemotherapy,” “radiation,” “fertility preservation,” “immune modulation,” and “neutrophil-mediated inflammation” were used to identify studies that explored neutrophils’ involvement in ovarian damage and repair during cancer treatment. Inclusion criteria for the selected studies included: original research articles (both clinical and preclinical studies), systematic reviews, and meta-analyses that focused on neutrophils in the ovarian microenvironment or their role in the context of cancer therapy and fertility. Only studies published in English were considered, and studies that did not focus on the relationship between neutrophils and ovarian function were excluded.

## Data extraction and synthesis

Data were extracted from the selected articles, focusing on key findings related to the impact of neutrophils on ovarian health, including their inflammatory effects, role in oxidative stress, and involvement in ovarian tissue repair. Special attention was given to the mechanisms through which neutrophils can contribute to ovarian damage, such as the release of cytokines, ROS, and proteases, as well as their reparative functions, including angiogenesis and the secretion of growth factors. Studies investigating therapeutic strategies aimed at modulating neutrophil activity in the ovarian microenvironment were also reviewed, with emphasis on pharmacological agents, immune modulation, and combination therapies with fertility preservation techniques. The data were organized into thematic sections that addressed the following: (1) the role of neutrophils in ovarian inflammation and damage during cancer treatments, (2) neutrophil-mediated repair mechanisms in the ovaries, (3) strategies to modulate neutrophil activity, and (4) the integration of these strategies into fertility preservation protocols. This thematic structure allowed for a coherent discussion of the current state of research and the identification of gaps in knowledge.

## Critical appraisal and analysis

Each study included in the review was critically appraised for methodological rigor and relevance to the topic. Studies were assessed for the quality of their experimental design, sample size, and the validity of their findings in relation to neutrophil function and fertility preservation. Discrepancies between studies, such as differences in findings regarding the effects of neutrophil recruitment on ovarian function, were discussed in the analysis, and potential reasons for these discrepancies – such as variations in cancer treatment protocols or patient populations – were explored. The findings were synthesized into a narrative that not only highlighted the key results from individual studies but also identified overarching trends, such as the emerging understanding of neutrophils as both detrimental and beneficial players in ovarian health. The review aimed to provide a balanced view of current research, suggesting potential future directions for clinical application and research.

## Neutrophils in the immune response and their role in cancer therapy

Neutrophils are the most abundant type of white blood cell and play a pivotal role in the innate immune response. They are the first responders to infections, rapidly migrating to sites of injury or infection to help combat pathogens through mechanisms such as phagocytosis, the release of antimicrobial proteins, and the formation of neutrophil extracellular traps (NETs). Despite their well-established role in immune defense, neutrophils are also involved in a range of pathological processes, including cancer development, progression, and response to therapy. Their ability to influence tumor microenvironments, both positively and negatively, has emerged as an area of significant interest in cancer immunology^[18,19]^. Neutrophils exert complex effects on the tumor microenvironment, where they can have both anti-tumor and pro-tumor activities. On one hand, neutrophils help control tumor growth through the release of cytotoxic molecules, such as ROS and proteases, which can damage cancer cells directly. In addition, neutrophils contribute to the activation of other immune cells, such as T cells, by presenting tumor antigens and secreting cytokines that promote an immune response against the tumor. However, the actions of neutrophils in the tumor microenvironment are often context-dependent. In some cases, neutrophils can promote tumor progression by creating a pro-inflammatory environment that supports angiogenesis, tumor cell survival, and metastasis. They may also suppress the activity of cytotoxic T lymphocytes and natural killer (NK) cells, which are key players in anti-tumor immunity^[20,21]^.

The role of neutrophils in cancer therapy is multifaceted and can vary depending on the type of therapy employed. In response to chemotherapy or radiation, neutrophils are often recruited to the site of tumor destruction, where they can exacerbate tissue damage or promote healing. In some cases, neutrophils can amplify the inflammation caused by these therapies, contributing to the development of therapy-related side effects such as fatigue, organ damage, and delayed recovery. Conversely, neutrophils can also enhance the therapeutic effects of cancer treatments by promoting tumor cell death and activating immune responses^[22,23]^. In addition to traditional cancer therapies like chemotherapy and radiation, newer approaches such as immune checkpoint inhibitors and targeted therapies also involve neutrophils. These therapies aim to enhance the body’s immune system to recognize and destroy tumor cells. Neutrophils, through their involvement in immune responses and their interaction with other immune cells, can either support or hinder the effectiveness of these therapies. For instance, neutrophils may enhance the activity of immune checkpoint inhibitors, which block inhibitory signals on T cells and NK cells, thus allowing a stronger immune response against tumors. However, they can also create a microenvironment that limits the effectiveness of these therapies by suppressing immune activation or promoting immunosuppressive pathways^[24–26]^.

Moreover, neutrophils are implicated in resistance mechanisms to cancer therapies. Tumors often exploit the immune system to evade treatment, and neutrophils can be part of this process. For example, in certain cancers, neutrophils can create a suppressive tumor microenvironment by secreting cytokines that inhibit the activity of immune effector cells. Additionally, neutrophils can contribute to the formation of a protective tumor niche, where cancer cells can survive and proliferate despite treatment^[27,28]^. The modulation of neutrophil function is an emerging strategy in cancer therapy. Several approaches are being explored to either enhance the anti-tumor activity of neutrophils or to limit their pro-tumor effects. For example, agents that promote neutrophil recruitment to the tumor site or enhance their cytotoxic activity could improve the efficacy of existing therapies. On the other hand, strategies to reduce neutrophil-mediated tumor progression, such as targeting pro-inflammatory cytokines or neutrophil-derived proteases, could help limit unwanted side effects and improve patient outcomes. By better understanding the complex role of neutrophils in cancer biology and therapy, it may be possible to develop more effective and personalized treatments for cancer patients^[29,30]^. Neutrophils are also involved in the context of oncofertility, where cancer treatments may damage reproductive organs, such as the ovaries. Neutrophils, through their inflammatory activities, can play a role in ovarian tissue damage during chemotherapy and radiation, leading to infertility in female cancer survivors. Oncofertility research aims to preserve fertility during cancer treatment, and understanding the role of neutrophils in ovarian function is essential. Neutrophils could either contribute to tissue damage or help with repair by promoting tissue regeneration. Their role in fertility preservation highlights their complex involvement in both cancer therapy and its side effects^[31–33]^.

## Neutrophils and ovarian function: a dual role in oncofertility

Neutrophils are the first responders of the immune system, playing a central role in host defense by combating infections and initiating inflammation. Their primary functions include phagocytosis of pathogens, the release of antimicrobial enzymes and ROS, and the formation of NETs that capture and neutralize harmful microorganisms. While neutrophils are essential for immune responses, emerging research has highlighted their involvement in other physiological processes, such as tissue repair and remodeling, particularly in the context of reproductive health. Neutrophils’ roles in ovarian function, especially during cancer treatment, are increasingly recognized as complex, with both beneficial and detrimental effects on fertility. This dual role is of particular importance in oncofertility, where preserving fertility during and after cancer therapy is a primary concern for many women^[34,35]^. Ovarian function is highly sensitive to changes in the immune microenvironment, and the infiltration of neutrophils into the ovaries can have significant implications for fertility. Chemotherapy and radiation therapies, commonly used to treat cancer, induce an inflammatory response that recruits neutrophils to ovarian tissue. These immune cells, in response to the damage caused by these treatments, can exacerbate ovarian damage through the release of pro-inflammatory cytokines, proteases, and ROS. The inflammatory environment created by neutrophils can lead to follicular atresia (the degeneration of ovarian follicles), oxidative stress, and alterations in the ovarian vasculature, ultimately contributing to reduced ovarian reserve and premature ovarian failure. These consequences are particularly concerning in the context of fertility preservation for cancer patients, as they can compromise the chances of successful conception^[36]^.

However, neutrophils also have a reparative role, which complicates their overall impact on ovarian function. In addition to their involvement in inflammation and tissue damage, neutrophils can aid in tissue repair through the secretion of growth factors such as VEGF and FGF. These factors can promote angiogenesis, tissue regeneration, and the healing of damaged tissue. In the ovarian microenvironment, neutrophils’ ability to enhance tissue repair and regeneration could mitigate some of the damage caused by chemotherapy and radiation. For instance, neutrophils may help repair ovarian stroma, restore blood flow, and support the regeneration of follicles, which are essential for maintaining fertility. This potential for neutrophils to aid in the recovery of ovarian tissue offers a promising avenue for improving fertility outcomes in women undergoing cancer treatment^[37]^. The dual role of neutrophils in ovarian function is influenced by various factors, including the type and timing of cancer treatment, the degree of ovarian damage, and the specific cytokine milieu present in the tumor and ovarian microenvironment. Chemotherapy regimens that rely on alkylating agents, such as cyclophosphamide, are particularly harmful to ovarian follicles, leading to significant fertility impairment. Neutrophils recruited to ovarian tissue following such treatments may exacerbate follicular loss and lead to irreversible damage. Conversely, more targeted therapies or lower-dose regimens may allow neutrophils to contribute to tissue repair and reduce the impact of chemotherapy on ovarian function. The complex interplay between neutrophils and ovarian tissue highlights the need for personalized approaches to oncofertility that consider both the immune and reproductive health of patients^[38]^.

One of the key challenges in oncofertility is the timing of intervention. Neutrophils are known to respond rapidly to tissue injury, and their early activation in the ovarian tissue during cancer therapy can significantly influence the extent of damage and recovery. Strategies aimed at modulating neutrophil activity during cancer treatment may offer a way to limit the negative effects of inflammation while promoting repair. For example, anti-inflammatory therapies that suppress excessive neutrophil activation or the use of agents that specifically target neutrophil-derived proteases could reduce ovarian damage. On the other hand, therapies that enhance neutrophil recruitment and activation at critical points in the treatment process could facilitate tissue regeneration and recovery, potentially improving fertility outcomes^[39–41]^. Beyond chemotherapy and radiation, other cancer treatments, including immune checkpoint inhibitors and targeted therapies, also influence neutrophil behavior. These therapies, which aim to boost the body’s immune response against cancer, may alter the dynamics of neutrophil infiltration into ovarian tissue. In some cases, these treatments could enhance the ability of neutrophils to promote anti-tumor immunity while also affecting ovarian function. Understanding how neutrophils interact with immune checkpoint inhibitors or other immune-modulating therapies could provide new insights into fertility preservation in cancer patients undergoing these newer treatments. Investigating the specific effects of neutrophils on ovarian function in response to these therapies is crucial for developing comprehensive strategies to protect fertility in patients receiving cutting-edge cancer treatments^[9,42–45]^.

## Neutrophils and the ovarian microenvironment

The ovarian microenvironment is an area where neutrophils have a complex and dual role, as their actions can both support and hinder ovarian function, particularly under the stress of cancer treatment^[46]^. The ovarian microenvironment is highly dynamic and essential for the regulation of reproductive health. It comprises various cell types, including granulosa cells, theca cells, oocytes, and stromal cells, all of which are involved in the regulation of ovarian function. In response to tissue injury or stress, such as that caused by chemotherapy or radiation, neutrophils are recruited to the ovaries, where they can have both protective and detrimental effects. Neutrophils influence ovarian health through the release of cytokines, growth factors, and proteases, which can either promote healing or exacerbate damage. Under normal physiological conditions, neutrophils are typically present in low numbers in the ovaries, but during pathological conditions, their presence increases significantly. This influx of neutrophils can modify the ovarian microenvironment in ways that affect ovarian reserve, fertility, and the overall functionality of the ovaries^[47]^. Neutrophils’ interaction with the ovarian microenvironment is primarily mediated through the release of pro-inflammatory cytokines such as interleukin-1 (IL-1), interleukin-8 (IL-8), and TNF-α. These molecules can recruit additional immune cells, including macrophages and T cells, to the site of inflammation, creating a cascade of immune responses. In the context of cancer treatment, chemotherapy and radiation can trigger the release of these inflammatory signals, exacerbating the effects on the ovaries. Neutrophils may contribute to follicular atresia, a process where developing follicles are prematurely eliminated, and this can lead to a significant reduction in ovarian reserve. The inflammatory cytokines and ROS released by neutrophils also cause damage to ovarian stromal cells and vasculature, leading to disruptions in the ovarian architecture and the ability to sustain normal reproductive function^[48,49]^.

One of the most concerning effects of neutrophil recruitment to the ovarian microenvironment during cancer treatment is oxidative stress. Chemotherapy and radiation therapy are known to increase the production of ROS, which can further activate neutrophils and trigger their release of additional ROS. This creates a vicious cycle of inflammation and oxidative stress, which can accelerate ovarian follicle depletion and increase the risk of ovarian failure. ROS also contribute to DNA damage in ovarian cells, further impairing ovarian function and potentially leading to long-term fertility issues. However, while ROS can damage ovarian tissues, they also play a role in cell signaling and tissue repair under normal circumstances, making the role of neutrophils in the ovarian microenvironment a double-edged sword^[50]^. On the other hand, neutrophils also have a reparative role that can be beneficial for ovarian tissue recovery. Neutrophils secrete a variety of growth factors, such as VEGF and FGF, which contribute to angiogenesis, tissue regeneration, and repair. These factors can help restore blood flow and promote healing of damaged ovarian tissue, facilitating the recovery of ovarian function following chemotherapy or radiation. Neutrophils can also aid in the removal of apoptotic cells, preventing the accumulation of dead cells and promoting tissue integrity. This reparative function of neutrophils in the ovarian microenvironment suggests that they might be beneficial in certain contexts, particularly when their activity is appropriately modulated to support tissue recovery without causing excessive inflammation^[51]^.

The balance between the inflammatory and reparative functions of neutrophils is influenced by various factors, including the type and timing of cancer treatment. For instance, chemotherapy regimens that involve alkylating agents, such as cyclophosphamide, are known to cause significant ovarian damage and inflammation. Neutrophils recruited to the ovaries in response to this damage may contribute to further follicle depletion and ovarian dysfunction. In contrast, lower doses of chemotherapy or more targeted treatments may allow neutrophils to play a more supportive role, helping to repair ovarian tissue and restore normal function. Understanding how neutrophils are activated and how they interact with the ovarian microenvironment during and after cancer treatment is crucial for developing therapeutic strategies that minimize ovarian damage and preserve fertility^[52]^. Recent studies have suggested that modulating neutrophil activity could be a potential therapeutic strategy for improving fertility outcomes in cancer patients. For example, inhibiting the release of pro-inflammatory cytokines or proteases from neutrophils might reduce the harmful effects of inflammation and oxidative stress on the ovaries. Additionally, promoting neutrophil-derived reparative functions, such as the secretion of growth factors, could enhance tissue recovery and reduce the risk of ovarian failure. Targeting neutrophil activity in a way that promotes healing while preventing excessive inflammation could be key to protecting ovarian function during cancer treatment^[53–55]^.

## Strategies to modulate neutrophil activity in oncofertility

The modulation of neutrophil activity represents a promising approach in oncofertility to protect ovarian function and preserve fertility in women undergoing cancer treatment. Neutrophils, as essential components of the immune response, play a dual role in ovarian health by both contributing to ovarian damage through inflammation and oxidative stress, and promoting tissue repair through growth factors and angiogenesis. By carefully regulating neutrophil activity, it may be possible to reduce harmful inflammatory responses while promoting ovarian regeneration. Several strategies have been proposed to modulate neutrophil activity in the context of oncofertility, ranging from pharmacological interventions to immune-targeted therapies. This article discusses the most promising strategies for modulating neutrophil activity to optimize fertility preservation in women undergoing cancer treatment^[56]^.

### Targeting neutrophil recruitment and activation

One of the primary mechanisms by which neutrophils contribute to ovarian damage is through their recruitment to inflamed ovarian tissue. Chemotherapy, radiation, and other cancer treatments induce an inflammatory response that recruits neutrophils to the ovaries. These immune cells then release pro-inflammatory cytokines, ROS, and proteases that can lead to follicular atresia and ovarian dysfunction. A strategy to prevent or modulate neutrophil recruitment could help mitigate the inflammatory damage to the ovaries. One approach is to target the chemotactic signals that attract neutrophils to the ovarian tissue. Molecules such as IL-8 and granulocyte-colony stimulating factor play key roles in neutrophil migration. Inhibiting these signaling pathways could reduce neutrophil infiltration into ovarian tissue during cancer treatment, thus preventing inflammation and oxidative stress from exacerbating ovarian damage. Other strategies could involve the use of small molecules or monoclonal antibodies that block specific receptors on neutrophils or the ovarian endothelium that mediate the recruitment process^[57]^.

### Inhibiting neutrophil-derived inflammatory mediators

Once neutrophils are recruited to the ovarian tissue, they release various inflammatory mediators, including pro-inflammatory cytokines (e.g., TNF-α, IL-1), ROS, and proteolytic enzymes such as neutrophil elastase and MMPs. These substances contribute to tissue damage, including the destruction of ovarian follicles and stroma. Inhibiting the release or activity of these inflammatory mediators could limit the negative impact of neutrophils on ovarian function. Several pharmacological agents are under investigation to inhibit neutrophil-derived inflammatory mediators. For example, neutrophil elastase inhibitors and MMP inhibitors have shown potential in reducing tissue destruction in other inflammatory conditions, and their use in the ovarian microenvironment could help reduce ovarian damage during chemotherapy. ROS scavengers, such as antioxidants, could also be employed to neutralize the damaging effects of ROS produced by neutrophils, potentially protecting the ovarian tissue from oxidative stress. Targeting these mediators can be a promising strategy for protecting ovarian reserve and fertility during cancer treatment^[56,57]^.

### Enhancing neutrophil-derived repair mechanisms

While neutrophils are primarily associated with inflammation and tissue damage, they also play a role in tissue repair. Neutrophils secrete growth factors such as VEGF and FGF, which promote angiogenesis, tissue regeneration, and healing. Enhancing these reparative functions could mitigate the damaging effects of cancer treatments on the ovaries and facilitate the recovery of ovarian function. One approach to enhance neutrophil-mediated repair is to stimulate the production of these reparative factors. For example, cytokines or small molecules that promote neutrophil activation and the release of growth factors could be used to encourage tissue healing in the ovaries after chemotherapy or radiation. Additionally, modulating neutrophil behavior to balance their inflammatory and reparative functions may provide a way to maximize their beneficial effects while minimizing damage. This dual approach of promoting tissue repair while controlling inflammation could hold great potential for fertility preservation strategies^[57]^.

### Use of anti-inflammatory agents to modulate neutrophil activity

Anti-inflammatory agents that suppress neutrophil activation or function may provide an effective means of protecting the ovaries from chemotherapy-induced damage. Nonsteroidal anti-inflammatory drugs, corticosteroids, and other immunosuppressive agents are widely used to modulate inflammatory responses in various medical conditions. These drugs work by inhibiting the production of pro-inflammatory cytokines and reducing neutrophil infiltration and activation. While these agents may help limit the harmful effects of neutrophils on ovarian function, their use in the context of fertility preservation must be carefully considered, as they could also impair the immune response to cancer. In particular, selective inhibition of neutrophil activation – without suppressing the overall immune system – may be a more targeted strategy. For example, drugs that block specific receptors involved in neutrophil adhesion (such as integrins) could prevent neutrophils from becoming activated and adhering to tissues in the ovaries. This targeted approach may reduce the inflammatory impact of neutrophils while preserving the body’s ability to fight infections and tumors^[58]^.

### Immunomodulatory approaches: tuning neutrophil function

Immunomodulatory therapies aimed at selectively tuning neutrophil activity offer a novel strategy for managing the complex immune interactions in the ovarian microenvironment. For instance, modulating the balance of Th1/Th2 cytokines or enhancing regulatory T cell (Treg) function could promote an anti-inflammatory environment in the ovaries, thereby limiting neutrophil-mediated damage. Treg cells play a role in suppressing excessive immune responses, and their induction or enhancement may help maintain ovarian homeostasis during cancer treatment. Additionally, harnessing the power of mesenchymal stem cells (MSCs), which have been shown to have immunomodulatory effects, may help control neutrophil activity. MSCs can secrete factors that influence the behavior of immune cells, including neutrophils, potentially reducing inflammation and supporting tissue regeneration. The combination of MSC therapy with traditional fertility preservation techniques may enhance the chances of restoring ovarian function after chemotherapy or radiation^[57,59]^.

### Targeting neutrophil-related signaling pathways

Neutrophils undergo activation through various signaling pathways, many of which can be targeted with pharmacological agents. For example, the nuclear factor kappa B (NF-κB) pathway, which is activated in response to inflammatory stimuli, plays a critical role in neutrophil activation and the release of pro-inflammatory cytokines. Inhibiting NF-κB signaling could reduce neutrophil-driven inflammation in the ovaries during cancer treatment, thereby protecting ovarian tissue from further damage. Other signaling pathways that regulate neutrophil function, such as mitogen-activated protein kinases, Janus kinase/signal transducer and activator of transcription, and the NLRP3 inflammasome, also represent potential targets for therapeutic intervention. By modulating these pathways, it may be possible to limit neutrophil-mediated damage to ovarian tissue while preserving their beneficial reparative functions^[60]^.

### Combining neutrophil modulation with fertility preservation techniques

Modulating neutrophil activity can complement existing fertility preservation techniques such as ovarian tissue cryopreservation, oocyte freezing, and ovarian transposition. These techniques aim to safeguard ovarian tissue from the toxic effects of chemotherapy and radiation, but they do not directly address the immune mechanisms that contribute to ovarian damage. By integrating neutrophil-modulating therapies with these fertility preservation strategies, the chances of maintaining ovarian reserve and fertility may be enhanced. For example, using anti-inflammatory agents to limit neutrophil recruitment during the critical period of chemotherapy or radiation could protect the ovaries while oocytes are preserved. Additionally, combining growth factors that promote tissue repair with ovarian tissue cryopreservation could support the recovery of ovarian function following treatment. Combining these approaches offers a holistic strategy to protect and restore fertility in women undergoing cancer treatment^[37,61]^.

### Personalized approaches to neutrophil modulation in oncofertility

The effects of neutrophils on ovarian function are influenced by factors such as the type of cancer, the chemotherapy regimen, and the patient’s individual immune response^[62]^. Personalized medicine approaches that tailor neutrophil modulation to the specific needs of each patient could optimize fertility preservation outcomes. Genetic profiling, immune cell analysis, and personalized treatment regimens could allow clinicians to select the most appropriate neutrophil-targeting therapies for each patient, minimizing adverse effects and maximizing ovarian protection^[33]^.

## Conclusion

Neutrophils, as key players in the immune response, hold a complex and dual role in oncofertility, particularly in the context of cancer treatments like chemotherapy and radiation. While their involvement in inflammation and tissue damage can contribute to ovarian dysfunction and fertility loss, their reparative functions offer a promising avenue for preserving ovarian health. By targeting neutrophil activity – whether through inhibiting their recruitment and activation, modulating the inflammatory mediators they release, or enhancing their tissue repair functions – it is possible to mitigate the negative effects of cancer treatments on ovarian reserve and fertility. Various strategies, such as immune modulation, pharmacological interventions, and combining neutrophil-targeted therapies with existing fertility preservation techniques, can help protect and restore ovarian function during cancer treatment. Personalized approaches that consider individual patient factors are likely to enhance the effectiveness of these strategies, providing a more tailored solution to preserve fertility in women undergoing cancer therapy. As our understanding of neutrophils in oncofertility deepens, future research is expected to identify more targeted and efficient methods to balance their inflammatory and reparative roles, offering new hope for women seeking to preserve their fertility while undergoing cancer treatment.

## Data Availability

Not applicable as this a review.

## Research registration unique identifying number (UIN)

Not applicable as this a review.
